# Integrin-Linked Kinase: It’s Role in the Vascular System

**Published:** 2007-03

**Authors:** Seung-Pyo Lee, Seock-Won Youn, Hyo-Soo Kim

**Affiliations:** *National Research Laboratory for Cardiac Stem Cell, Seoul National University College of Medicine, Seoul, Korea*

**Keywords:** integrin-linked kinase, vascular biology, blood vessel, endothelium

## Abstract

Integrin-linked kinase (ILK) is an intracellular molecule that binds to the cytoplasmic domain of β1 and β3-integrin. It has been previously demonstrated in various epithelial cell lines to mediate the ‘outside-in’ signals into the cells and to control the survival of these cells by controlling the phosphorylation of various downstream proteins, such as protein kinase B/Akt (PKB/Akt). We now present in this review the important role of ILK in the vascular system with particular emphasis on its role in endothelial cells (ECs). The results presented here demonstrate that ILK is essential for the proper function, structure and survival of ECs and finally, for the process of neovascularization.

## INTRODUCTION

Coronary artery disease and cerebrovascular disease are devastating status of acute vessel occlusion in diseased vessels that are already narrowed enough by atherosclerotic process. According to a Korean data, this disease account for more than 50% of the total cause of death and the burden continues increase. These diseases are caused basically by ischemic damage of the target organ, for example acute myocardial infarction from coronary artery and ischemic stroke from intracerebral artery occlusion. The prevention of these cardiovascular diseases involves the meticulous use of multiple drugs such as aspirin and beta-blockers, but these drugs are not enough to regenerate the organ or tissue that has been already damaged by preceding vascular events. People are now focused on tissue regeneration (1) and one method to do so is the supply and growth of new vessels into the ischemic tissue. It is now well-known that blood vessels are not only a “passive conduit” for passage of blood components and gas, but an “active participant” of several physiological/pathological processes that we encounter in everyday life in the clinics.

Integrin-linked kinase (ILK) is an intracellular molecule that has been under intense investigation nowadays. Several groups, including our laboratory, have focused on its role in the vascular system in the past couple of years, and have demonstrated that the role of ILK is not only important in tumorigenesis in several epithelial cells but that it is also a critical molecule mediating the survival and function of endothelial cells (ECs). It has also been demonstrated that not only is ILK important in ECs but it is also critical in the function (2) and recruitment of endothelial progenitor cells (EPCs) to the appropriate tissue (3).

Therefore ILK provides a new, interesting target of genetic modulation in either enhancing or suppressing the process of neovascularization. Unraveling the natural role of ILK in the vascular system is expected to be a cornerstone in this field. In this review, we will introduce the role of ILK in the vascular system, together with the results of the work in our laboratory, which has lead to some interesting results concerning the role of ILK in EPCs as well.

### Integrin-linked Kinase: Its discovery as a both structural and catalytic protein in epithelial cells

ILK was first discovered at 1996 by Hannigan and Dedhar as an intracellular molecule that binds to the cytoplasmic domain of β1 and β3-integrin and is expressed ubiquitously in various tissues and cells (4). It is mainly comprised of 3 structurally different regions (5). There are 4 ankyrin (ANK) repeats in its N-terminus and then a pleckstrin homology (PH)-like motif. Lastly, there exists a domain at the C-terminus which shares significant homology with other protein catalytic domains. The concern on ILK was initially focused on its ability to mediate various protein-to-protein interactions and also its ability to transduce the external mechanical stimuli to an internal chemical stimulus.

Up to date, there are 10 proteins that can bind to ILK and these can be largely divided into 3 parts (5). The list of these proteins is listed in Table 1. The first and second sets of proteins are adaptor proteins and integrins that connect the extracellular matrix with the intracellular structural proteins such as actin cytoskeletons. Particularly interesting Cys-His rich motif (PINCH), a focal adhesion protein, and the ANK1 domain of ILK binds to each other to maintain the cell shape (6, 7). PINCH is comprised of 5 LIM domains (8) and it has been shown previously that it can undergo considerable conformational change upon binding of ILK (9). In addition, PINCH binds to various receptor tyrosine kinase via Nck2 (10). *In vivo*, PINCH is required for the assembly of muscle-dense bodies in studies of *Caenorhabditis elegans (C. elegans)* (11). Disruption of ILK in Drosophila lead to the detachment of actin filaments from the muscle attachment site, suggesting that the PINCH-ILK interaction is indeed important for maintaining the structure of the cells *in vivo* (12).

**Table 1:** ILK-binding proteins

ILK-binding protein	Binding site on ILK - binding site on ILK- binding protein
β1-integrin	kinase domain - cytoplasmic domain of β1-integrin
β3-integrin	undetermined
PINCH	ANK1 domain - LIM1 domain
ILKAP	ANK1 domain - undetermined
PDK-1	kinase domain - undetermined
PKB/Akt	kinase domain - undetermined
α-parvin	kinase domain - CH2 domain of parvin
β-parvin	kinase domain - CH2 domain of parvin
γ-mparvin	unknown - CH2 domain of parvin
Mig-2	kinase domain - undetermined

Parvins, that consists of 3 set of proteins, α-parvin/actopaxin/calponin homology ILK binding protein (CH-ILKBP), β-parvin/affixin and γ-parvin, is a protein that binds to the kinase domain of ILK (13-16) and acts as a critical protein maintaining the cell shape and mechano-transduction into the cell (17). α-parvin/actopaxin/CH-ILKBP interacts directly with actin, as well as indirectly by binding with paxillin (13, 15). β-parvin can also link ILK with actin by interacting with α-actinin (18). The proteins that interact with γ-parvin are not identified yet.However, there has been interesting, arguing results on the role of γ-parvin in hematopoietic cells. Although the purpose and the viewpoints of the experiments were very different, deletion of γ-parvin *in vitro* has been shown to result in defective monocyte migration and spreading to ECM (19), whereas disruption of γ-parvin *in vivo* lead to no gross abnormality in the development of hematopoietic system (20).

Mitogen inducible gene-2(Mig-2), a mammalian homolog of UNC-112 in *C. elegans*, is also a molecule that interacts with the filaminin component of actin (21). Mig-2 has also been shown to be a candidate molecule linking actin to ILK (22). In an experimental model of others, ILK was required for the proper localization of structural proteins to integrin foci, suggesting that ILK is a critical protein mediating outside-in and inside-out signals in cells (22).

**Figure 1:**
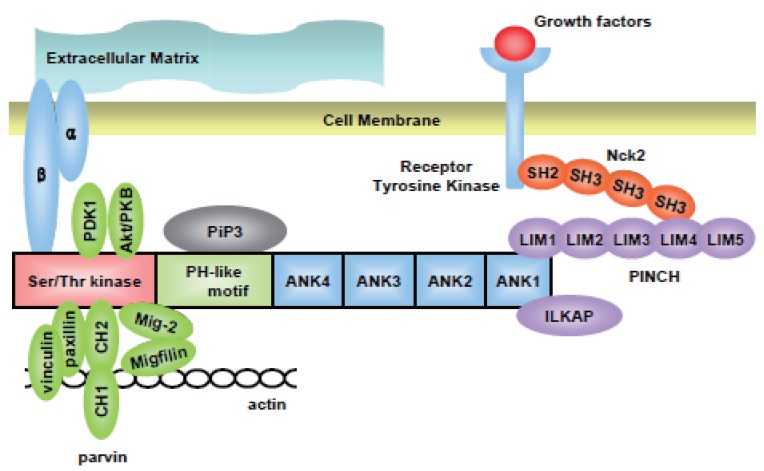
Interaction of Integrin-linked Kinase (ILK) with various intracellular proteins. ILK is made up of 3 distinct domains with 4 ankyrin (ANK) repeats at the N-terminal, Ser-Thr kinase domain at the C-terminal and the pleckstrin homology (PH) motif in between these two domains. Particularly INteresting Cys-His rich protein (PINCH), which consists of 5 LIM domains interacts with ANK1 of ILK is also bound to various receptor Tyr kinase via another adaptor protein Nck2. ILK associated protein (ILKAP) is another protein that binds to the ANK1 of ILK, which functions to inhibit the kinase activity of ILK. Phosphatidylinositol-3, 4, 5-triphosphate (PiP3) is a product of phosphoinositide-3-kinase (PI3-kinase), that is thought to bind to the PH domain of ILK. The kinase domain of ILK binds to several intracellular proteins including the cytoplasmic domain of β1 and 3 integrin. It also binds to numerous kinase proteins such as phosphatidylinositol-3-kinase-dependent kinase-1 (PDK-1) and protein kinase B (PKB)/Akt. Parvins, which is consisted of 2 calponin homology (CH) domain, links ILK directly to actin or indirectly by interacting with paxillin and vinculin. Mitogen inducible gene-2 (Mig2) is another new protein that has been discovered to bind to ILK. It also has the potential to bind to actin by interacting with migfilin.

The third set of molecules are catalytic proteins, such as PKB/Akt (23) and ILK associated protein (ILKAP) (24). The kinase activity of ILK is under regulation of certain proteins such as ILKAP (24) and phosphoinositide-3-kinase (PI3-kinase) (25) and this peculiar kinase activity of ILK needs PH motif, as mutants of the PH-domain in ILK leads to the inability of ILK to phosphorylate Akt/PKB (26). Several proteins that directly interact with ILK are demonstrated in Figure 1.

Together with these structural functions of ILK, the catalytic function of this protein has also been under in-tense investigation until recently. The role of ILK in various epithelial cells has been shown by analyzing the ILK activity in either growth factor or extracellular matrix (ECM) stimulated cells, or in ILK-overexpressing cells. Overexpression of ILK in various epithelial cell lines has been shown to activate PKB/Akt (26), mitogen activated protein kinase (MAP kinase) (27) and inactivate glycogen synthase kinase 3β (GSK-3β) (25, 28). Moreover it has also been shown to downregulate E-cadherin (29, 30) and activate the nuclear translocation of β-catenin (30), possibly by inactivating GSK-3β. Cyclin D1 expression has also been shown to be upregulated by activation of activating protein-1 (AP-1) through inactivation of GSK-3β (28, 31). But these trafficking of intracellular proteins are not always consistent, as the activation/inactivation of Akt, GSK-3β and β-catenin does not take place in Drosophila system after ILK overexpression (32), suggesting that the downstream molecules in one type of cells are not so in another.

**Figure 2:**
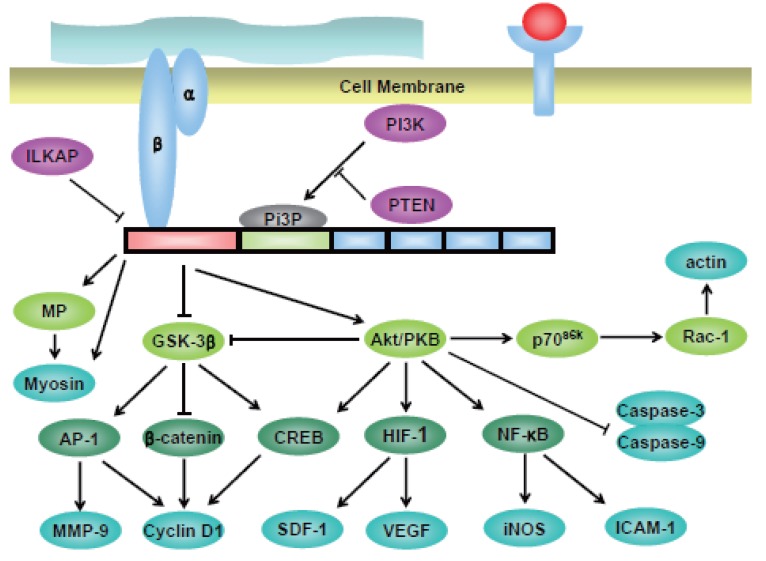
Signaling pathways involving ILK. The catalytic activity of ILK is positively regulated by PI3-kinase which functions to activate PKB/Akt and inhibit glycogen synthase kinase 3β (GSK-3β). PI3-kinase is also involved in the interaction of parvin with ILK. Conversely, ILK is negatively regulated by two proteins; phosphatase and tensin homolog deleted on chromosome 10 (PTEN) and ILKAP. PTEN is mainly involved in the phosphorylation of the Ser-473 residue of PKB/Akt and thus, activation of it, whereas ILKAP is involved in the phosphorylation and inhibition of GSK-3β. ILK phosphorylates and activates PKB/Akt. This, in turn, activated several transcription factors such as cAMP-response element binding protein (CREB), hypoxia-inducible factor-1 (HIF-1), nuclear factor-κB (NF-κB). Also, PKB/Akt can suppress various caspases, such as caspase-3 and 9. These factors can activate numerous effector molecules involved in cell cycle progression, inflammation and angiogenesis. Also PKB/Akt can be involved in cell morphogenesis and migration by rearrangement of actin filaments. ILK can also phosphorylate and inactivate GSK-3β directly or indirectly by pathways involving PKB/Akt. The inactivation of GSK-3β leads to the activation of cyclic adenosine monophosphate response element-binding (CREB), activating protein -1 (AP-1) and also inactivation of β-catenin. These are the key factors controlling cell invasion into the extracellular matrix and cell cycle progression/cell proliferation. Also, ILK regulates muscle contraction by directly phosphorylating myosin and also, indirectly via myosin light-chain phosphatase (MP).

One of the functions that has been of particular interest in ILK is its activity to suppress apoptosis and anoikis (suspension-induced apoptosis) (23, 33). These effects are mainly due to the suppression or activation of proteins that are involved in cell survival and apoptosis such as PKB/Akt (25) and caspase-3 and 8 (33). It has been demonstrated in mammary epithelial cell lines that overexpression of ILK leads to the suppression of anoikis in a PKB/Akt-dependent manner (33) and these phenomenon has also been shown to occur in various cancer cell lines such as breast cancer (27) and prostate cancer (23). The overall substrates of ILK are depicted in Figure 2.

Taken together, these findings indicate ILK as a unique protein that can act as both structural and catalytic protein in epithelial cells. These findings have been adopted and utilized in the field of oncology and ILK holds as a promising target of many neoplastic disease such as prostate cancer and breast cancer (34).

### The discovery of ILK in the vascular system

ILK has been under intense investigation in various cell lines in epithelial lineages but the role of ILK in the vascular system has been under investigation very recently. This part of the paper would focus on the role of ILK in vascular biology with particular emphasis on its role in ECs and the process of neovascularization.

**1) The role of ILK in endothelial cells.** The possibility of the role of ILK in ECs, has first been known in 2001 by Zhang *et al*., who demonstrated that oxidized LDL (ox-LDL) treatment in human umbilical vein endothelial cells (HUVECs) resulted in the transcriptional and translational upregulation of ILK (35). The overexpression of ILK lead to the suppression of ox-LDL-induced apoptosis and this phenomenon was mediated by various intracellular molecules involved in apoptosis and survival, such as p38 MAP kinase, bcl-2, bcl-xL, caspase-3 and 9. These findings shed light into the possibility that the pathways found in epithelial cell lineages would also happen in ECs and that genetic enhancement of ILK in ECs would be protective. This possibility was partially confirmed by Gonzalez-Santiago *et al*. shortly afterwards, who showed that the HUVECs cultured in collagen type I, a pathologic condition in which HUVECs are not ordinarily exposed to, showed decreased endothelial nitric oxide synthase (eNOS) activity and also, decreased ILK kinase activity (36). This phenomenon was rescued by overexpression of ILK in collagen type I cultured HUVECs, thus confirming that ILK could act as a molecule in reducing various stress. The ability of ILK to rescue HUVECs under specific stress, such as oxidative stress and abnormal ECM, was further expanded to conditions such as starvation (nutrient deprivation) and cell suspension (anchorage deprivation) in another paper from our group (2). The authors took the advantage that ILK was transiently upregulated after nutrient and anchorage deprivation in HUVECs and that enhancing the natural increase of ILK would naturally lead to the suppression of apoptosis under these stressful conditions. These effects of ILK was partially dependent on several intracellular molecules, such as PKB/Akt and GSK-3β, previously described to be downstream of ILK in various epithelial cells, supporting that many of the intracellular pathways found in the mammalian epithelial cells would also occur in ECs as well.

Another part of the role of ILK in ECs mainly stem from the idea that ILK is upstream of a well-known intracellular molecule PKB/Akt, which in turn is known to be important in the function and survival of ECs *in vitro* and *in vivo*. Also, vascular endothelial growth factor (VEGF), a strong mitogen and survival factor in ECs, is known to be secreted in various cancer cells such as prostate cancer (37) and breast cancer (38, 39), which in turn activates PKB/Akt in ECs (40, 41). Therefore it is logical to think that ILK would be important in the intracellular signaling and the function in ECs. In accordance to this hypothesis, the paper by Tan and Dedhar shows exactly the critical importance of ILK in ECs. According to this paper, prostate cancer cells secrete VEGF in a ILK-dependent manner via pathways involving Akt→m-TOR (mammalian target of rapamycin)→HIF-1 (hypoxia inducible factor-1) (42). Also, the functions of ECs by the secreted VEGF were assessed by survival, migration and invasion assay and also by tube formation and EC sprouting assay. This action of VEGF in ECs was completely abrogated by blockade of ILK with transfection of si-RNA of ILK, confirming that the above functions of VEGF in ECs were ILK-dependent. The aforementioned action of VEGF via ILK was again confirmed by the papers from Kaneko and Basaki, published shortly afterwards. ILK was found to be bound to VEGFR-2 in response to VEGF stimulation and the main functions of VEGF was suppressed by KD-ILK (kinase deficient form of ILK) transfection (43). These phenomena emphasize the kinase function of ILK in maintaining the basic function of ECs and that ILK is a critical molecule mediating the VEGF→PKB/Akt pathway.

However, it was shortly followed by another paper by Vouret-Craviari and van Obberghen-Schilling, who demonstrated that ILK is important in EC adhesion and capillary formation (44). This role of ILK was associated with distribution of α5β1 integrin on the cell surface and also with fibrillar fibronectin matrix. Interestingly, silencing ILK with RNA interference was shown to induce morphological changes in the structure of ECs, changing the shape of EC to a rather round shape. Also, silencing of ILK induced the cellular expression of certain integrins, such as α5β1 and αvβ3 integrin, and finally, increased cell adhesion to ECM, whereas cell spreading, migration and tube formation was impaired in ILK-depleted cells. In accordance with these findings, Watanabe and Basaki also noticed that transfection of KD-ILK plasmid resulted in decreased tube formation in HUVECs stimulated with VEGF (45). Therefore, one can implicate from the above findings that ILK is indispensable in the dynamic features of ECs, such as proliferation, migration and tube formation and that the kinase function of ILK is more important in these features than its structural association with other intracellular proteins.

The natural role of ILK in EC was further implicated to *in vivo* findings by other groups which lead to some very interesting findings. Friedrich and Gerszten were among the first to find that conditional knock-out of ILK in ECs leads to a defect in embryogenesis by placental insufficiency caused via decreased labyrinthine vascularization *in vivo* and massive apoptosis of ECs *in vitro* (46). But discordant to the previous findings by Vouret-Craviari and van Obberghen-Schilling, deletion of ILK in ECs resulted in a characteristic detachment of ECs from the matrix and loss of actin fibers. Whether the discrepancy between these two independent experiments is due to the experimental conditions or the technique used to knock-out ILK (si-RNA vs. Cre-recombinase conditional knock-out) remains to be confirmed. The finding in this paper is concordant with another previous paper, in which ILK knock-out resulted in a lethal phenotype, mainly by a defect in actin rearrangement but also defective vascular development (47).

**Figure 3:**
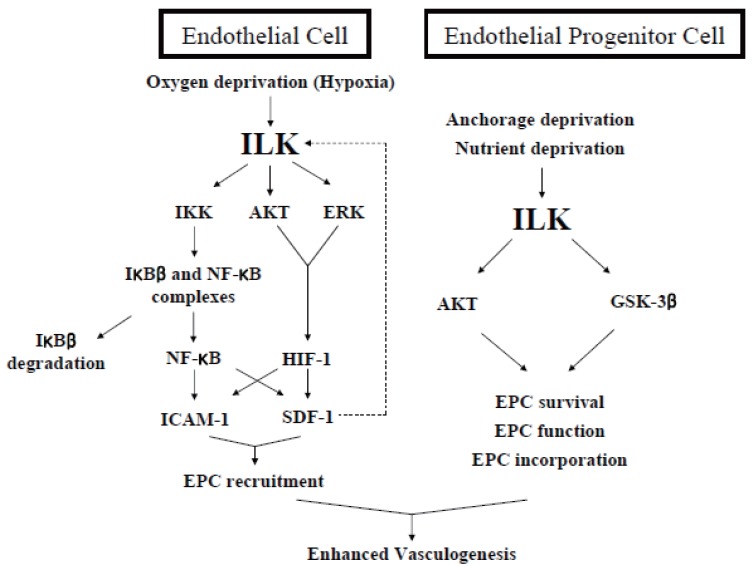
Role of ILK in postnatal vasculogenesis. ILK has roles both in endothelial cells (ECs) and endothelial progenitor cells (EPCs), the two utmost critical cells involved in the process of vasculogenesis. First, it acts to recruit EPCs selectively to ischemic tissue by enhancing the expression of stromal cell derived factor -1 (SDF-1) and intercellular adhesion molecule -1 (ICAM-1) on the surface of hypoxic ECs. Second, ILK is a critical molecule in EPC survival, function and incorporation. Taken together, ILK functions both in ECs and EPCs to enhance vasculogenesis *in vivo*.

In addition to the previous works to reveal the basic role - especially the structural function - of ILK in ECs, efforts to control the process of vasculogenesis by genetic modulation of ILK has also lead to some interesting, new findings mainly from our laboratory. The previous findings in our laboratory that ILK is transiently elevated in various stress conditions in ECs also held true in EPCs from adult human peripheral blood (2) and this phenomenon has given the idea that enhancing ILK would also reinforce the survival pathway involving PKB/Akt, GSK-3β and finally vasculogenesis. *In vitro*, ILK-overexpressing EPCs showed superior ability in incorporating into the EC layer and this was successfully implicated to the *in vivo* model. ILK-overexpressing EPCs were successful in salvaging ischemic tissue even with only 5% of the cells given in the control group. This finding has the major ability to overcome the obstacle in the sparcity of EPCs in adult human peripheral blood by genetic modification. Together with the findings that ILK has a critical role in EPC function, it was also demonstrated in the following paper from our laboratory that ILK was also critical in EPC recruitment to the appropriate ischemic tissue. ILK was naturally enhanced in its amount and also in its kinase activity in hypoxic ECs. The abrogation of the kinase activity of ILK in hypoxic ECs lead to a significantly suppressed expression of stromal cell derived factor -1 (SDF-1) and intercellular adhesion molecule -1 (ICAM-1), the two key molecules found to be important in EPC recruitment to ischemic tissue (48, 49). Finally, genetic engineering of ILK in hypoxic tissue lead to the control of EPC recruitment in ischemic tissue and finally vasculogenesis. These findings of the important role of ILK in postnatal vasculogenesis can be summarized into Figure 3.

ILK has also been found to have a potential role in EC dysfunction. Although there is limited data on this subject, it has been demonstrated rather earlier that ILK is involved in EC-ECM interaction and endothelial dysfunction. Seeding EC on collagen I rather than a normal ECM, collagen IV, decreased the expression of eNOS and also the production of nitric oxide from ECs, the effect of which was reversed by overexpression of ILK (36). The aforementioned control of eNOS by ILK was not further investigated in depth but one can guess that both the kinase activity (activation of PKB/Akt by ILK, which in turn can activated eNOS (50, 51)) and the structural function (maintenance of actin cytoskeleton by ILK, which in turn regulates eNOS activity (52)) of ILK can be involved in this process. This finding indicates that ILK can be another new, biologic marker of endothelial dysfunction. Together with the previous data, it has also been shown that culture of EC in collagen I leads to decreased activity of ILK and also increased expression of MCP-1 and adhesion of peripheral blood monocytes to ECs (53). This effect was abrogated by transfection of ILK into EC, which also suggests that ILK is involved in endothelial dysfunction. Whether these *in vitro* findings have further implication into *in vivo* findings, such as atherosclerotic disease, also remains to be confirmed.

**2) The Role of ILK in Platelets.** Although there is a limited amount of data in this field, the role of ILK in platelets has also been demonstrated in studies done in platelet activation. The potentiality of the involvement of ILK in this process is a fancy target of research, concerning that ILK is a intracellular protein that binds to β1 and β3-integrin, the two key surface molecules involved in inflammation and platelet activation. Although the experimental methods and design differ slightly from each other, a few groups have commonly demonstrated that the kinase activity of ILK increases after stimulation of platelets (54-56). Moreover, ILK has been shown in platelets to mediate the ‘activation’ signal into the cell - for example, activation of platelets by adenosine diphosphate or thrombin - and in turn, to ‘outside’ activity - for example, increased phosphorylation of β-integrin. These effects have been confirmed and further implicated into the adhesion activity of platelets (57). Platelets activated by thrombin exhibited increased kinase activity of ILK and ultimately, to increased adhesion to collagen. Therefore, the role of ILK may be further implicated to *in vivo* models of acute thrombosis and this remains to be further investigated.

**3) The Role of ILK in Vascular Smooth Muscle Cells.** In contrast to the well-investigated role of ILK in ECs, its role in vascular smooth muscle cells (VSMCs) is largely not understood. Specifically, there has recently been one journal reporting that the amount and activity of ILK is specifically upregulated by angiotensin-II and downregulated by statin treatment *in vitro* and *in vivo* (58). This paper implicates that ILK may play a critical role in atherosclerosis and that it would be a good potential target of anti-atherosclerotic management. As in the role of ILK in ECs, the investigation of its role in VSMCs is further warranted in the future.

**Figure 4:**
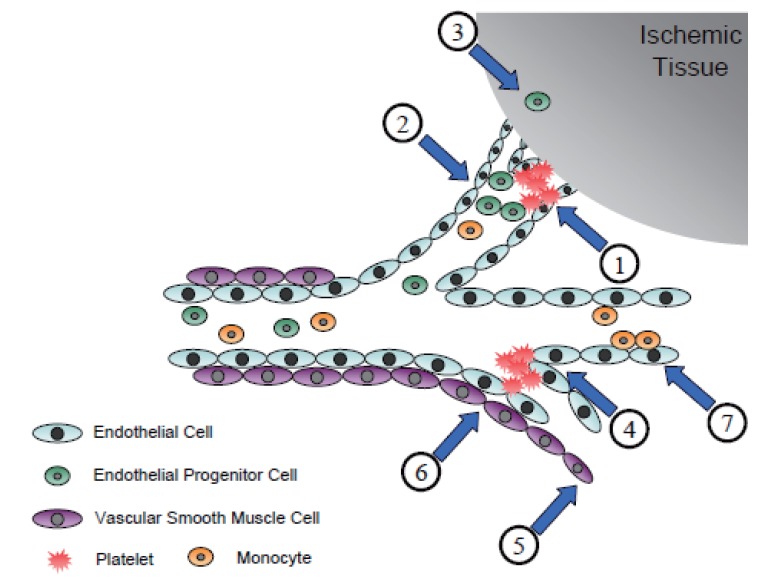
Role of ILK in the vascular system. ILK has been found to be involved in a variety of cells composing the vascular system. 1) ILK is thought to mediate the ‘outside-in’ signals in activated platelets. The activity of ILK and the association of this ILK with β-integrin is highly enhanced after the activation of platelets. This, in turn, is thought to enhance the ability of aggregation, which remains to be a controversy yet. 2) ILK is also activated in hypoxic endothelial cells. The activated ILK mediates the expression of SDF-1 and ICAM-1, which in turn is critical in endothelial progenitor cell recruitment to the ischemic tissue. Thus, ILK is thought to control the proper homing of endothelial progenitor cells. 3) ILK is critical in neovascularization to the ischemic tissue, either by mediating the sprouting of ischemic endothelial cells, per se (angiogenesis), or by controlling the function and survival of the recruited endothelial progenitor cells (vasculogenesis). The role of ILK still remains to be investigated in the vascular biology, such as the following. 4) It remains to be investigated whether ILK has any role in the interaction between activated platelets and endothelial cells. 5) The role of ILK in vascular smooth muscle cells, another major component of the vascular system, remains largely unknown. 6) Whether ILK has any effect on endothelial cell-vascular smooth muscle cell interaction is also unknown. 7) Finally, the role of ILK in the interaction between endothelial cell-monocyte interactions, which is well-known to be important in the initiation of atherosclerosis, is another unknown project.

Taken together, the role of ILK in the vascular system that has been verified and also, its role that needs further study, can be summarized into Figure 4.

## CONCLUSION

ILK is an intracellular protein that is ubiquitously expressed in various types of cells and tissues. As its role in several neoplastic diseases is now being revealed, it is also known to be a key molecule also in the vascular biology, particularly ECs. But, it remains to be further investigated whether it has any role in other components of the blood vessel, such as vascular smooth muscle cells. In addition, many of the findings of ILK in this field have been focused on *in vitro* findings but its role in various *in vivo* models is very limited. Together with the forecoming results of ILK in various human disease models, we hope to gain an integrated understanding of the role of ILK in vascular biology.
